# Medical undergraduates’ self-evaluation: before and after curriculum reform

**DOI:** 10.1186/s12909-022-03330-w

**Published:** 2022-04-20

**Authors:** Jeng-Cheng Wu, Kung-Pei Tang, Yi-Hsin Elsa Hsu, Ya-Ting Yang, Jan-Show Chu, Yen-Kuang Lin, Wen-Hsuan Hou

**Affiliations:** 1 Department of Urology, Taipei Medical University Hospital, Taipei, Taiwan; 2 Department of Urology, School of Medicine, College of Medicine, Taipei Medical University, Taipei, Taiwan; 3 Department of Health Promotion and Health Education, College of Education, National Taiwan Normal University, Taipei, Taiwan; 4 National Taipei University of Education, Taipei, Taiwan; 5 International Ph.D. Program in Biotech and Healthcare Management, Taipei Medical University, Taipei, Taiwan; 6 Center for General Education, Taipei Medical University, Taipei, Taiwan; 7 School of Medicine, College of Medicine, Taipei Medical University, 250 Wu-Hsing Street, Taipei, 11031 Taiwan; 8 Graduate Institute of Athletics and Coaching Science, National Taiwan Sport University, Taoyuan, Taiwan; 9 Biostatistics Research Center, Taipei Medical University, Taipei, Taiwan; 10 Department of Physical Medicine and Rehabilitation, Taipei Medical University Hospital, Taipei, Taiwan; 11 Department of Geriatrics and Gerontology, Taipei Medical University Hospital, Taipei, Taiwan; 12 School of Gerontology and Long-Term Care, College of Nursing, Taipei Medical University, Taipei, Taiwan; 13 Graduate Institute of Clinical Medicine, College of Medicine, Taipei Medical University, Taipei, Taiwan

**Keywords:** Curricular reform, Student preparedness, Curriculum, Undergraduate medical education

## Abstract

**Background:**

In 2013, Taiwan launched a curriculum reform—the 7-year undergraduate medical education program was shortened to 6 years. This study explored the evaluation results from students regarding the curriculum reform and investigated graduates’ perceptions regarding the curriculum organization of the two academic training programs affected by this curricular reform.

**Methods:**

A cross-sectional survey was conducted from May 14 to June 12, 2019. The 315 graduates from both the 7-year and 6-year curriculum programs in the same medical school in Taipei were invited to participate in this study. In total, 197 completed questionnaires were received, representing a response rate of 62.5%. The results of the principal component analysis confirmed the validity of the constructs employed in this self-administered questionnaire.

**Results:**

The *t*-test results yielded two main findings. First, the graduates from the 6-year program had significantly lower scores for preparedness for the upcoming postgraduate-year residency training than did their 7-year program counterparts. Additionally, the male graduates had significantly higher scores in terms of perceptions regarding curriculum organization and preparedness for postgraduate-year residency training than the female graduates. The results of stepwise regression also indicated that the sex difference was significantly correlated with graduates’ readiness for their postgraduate-year residency training.

**Conclusion:**

To avoid sex disparities in career development, a further investigation of female medical students’ learning environment and conditions is necessary. In addition to the cross-sectional study of students’ perceptions, further repeated measurements of the objective academic or clinical performance of graduates in clinical settings are desirable.

## Background

According to Kirkpatrick’s model, the most direct evaluation of a training program is the participants’ feedback [1]. Therefore, medical education entities have relied on students’ evaluations to measure the quality and effectiveness of their educational practices and programs [2–7]. Lockwood et al. and Pugnaire et al. used questionnaires to survey the graduates of the Association of American Medical Colleges and discovered that students’ perceptions of their medical program were consistent and reliable [8, 9]. Schools have even been able to use students’ input in the classroom environment to predict their learning outcomes [10, 11]. With reference to questionnaires such as the Dundee Ready Educational Environment Measure or Undergraduate Clinical Education Environment Measure, which assess interpersonal interactions and social factors within medical educational environments, we developed a questionnaire that only focuses on students’ views on their previous academic learning as well as the upcoming training program [4, 12, 13]. Other Taiwanese medical educators, such as Chan et al., have also collected students’ feedback on their satisfaction rate in terms of their confidence in their medical education through surveys to improve the training programs’ quality [14]. In the survey results from three countries—the United States, Australia, and Taiwan—medical students exhibited similar satisfaction rates (i.e., 70.7%–86.6%) toward their training curricula. However, the self-confidence of Taiwanese students (55.9%) regarding participation in a residency program was markedly lower than that of American students (88.6%), which might indicate the insufficiency of Taiwanese medical students’ clinical training [14].

Chan’s survey was conducted prior to the medical program reform in Taiwan. At that time, the medical schools in Taiwan offered a 7-year program leading to the awarding of a Doctor of Medicine (MD) degree in the direct entry system format. The 7-year curriculum included 2 years of premedical courses, 2.5 years of clinical courses, and 2.5 years of clerkship and internship training. Students were required to attend clinical courses in hospitals for a minimum of 3 days per week in years 5 and 6 of their training. Year-7 students participated in a full-time internship to receive placement training while performing clinical procedures and examinations on real patients under the supervision of senior staff [14, 15].

During the 2003 severe acute respiratory syndrome epidemic in Taiwan, many Year-7 medical students were assigned as first responders alongside postgraduate-year (PGY) residents to accommodate the urgent demands of the workforce. This experience revealed some curricular shortcomings of the medical training programs in Taiwan, leading to calls for reform in the field. In particular, the previous curriculum aimed at training medical specialists at the beginning of the postgraduate training year instead of providing sufficient clinical training in terms of general medicine [16]. An initial phase of reform was subsequently undertaken to focus on general medicine training in the postgraduate years [17].

**Fig. 1:**
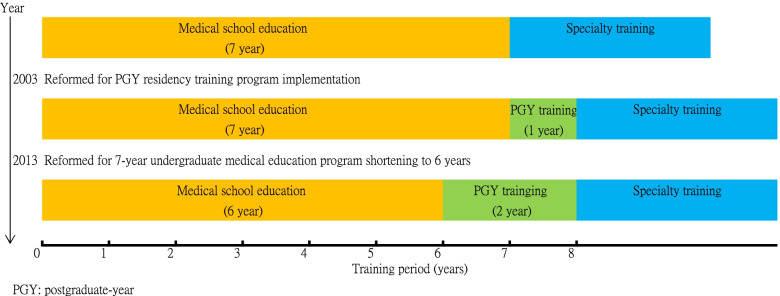
Development of Undergraduate Medical Education and Professional Training Program for 2000–2020 in Taiwan

In 2013, the 7-year undergraduate medical education program in Taiwan was shortened to 6 years to implement a complete 2-year PGY residency program following undergraduate medical training [15, 16]. Because of the rapid development of medical technology and changes in the medical environment, medical education reform is a major global concern [18]. Successful experiences in medical education reform in Western countries have been widely disseminated; however, they may not be directly applicable to Asian countries because of differences in social and cultural dispositions [19]. Taiwan’s curriculum reform adopted the concept of a foundation program in the United Kingdom and was officially launched in 2013 and immediately implemented in all medical schools (see Fig. 1) [20]. The initiation and process of medical education reform in Taiwan has been discussed previously [21]; however, no difference was observed in the national Objective Structured Clinical Examination scores between 6-year and 7-year curriculum graduates [22]. In 2019, the medical field welcomed the last graduates of the 7-year training program and the first graduates of the 6-year training program since the reform. In this study, we compared students’ feedback on the quality and effectiveness of each curriculum system to consider students’ perceptions of which system better prepares them for postgraduate training.

## Methods

### Participants and procedures

A cross-sectional survey was conducted in the spring of 2019 to 315 students who graduated from the two curriculum systems of the same medical school in Taipei. After providing signed informed consent, the participants completed a self-administered questionnaire during their learning feedback meetings before graduation.

### Scale

To align with the general competency domains of the Accreditation Council for Graduate Medical Education (ACGME), which is widely adopted to frame medical education objectives in Taiwan, we embedded the following six domains in the questionnaire: patient care, medical knowledge, practice-based learning and improvement, interpersonal and communication skills, professionalism, and system-based practice [17, 23]. A 5-point Likert scale (from 1 = strongly disagree to 5 = strongly agree) was used for students to evaluate items pertaining to the first level of Kirkpatrick’s four-level training evaluation model. Data on the other levels were not available and thus were not included.

In addition to the demographic variables (sex/age/year of graduation), the design and development of this questionnaire incorporated Kirkpatrick’s model and Azjen’s theory. The first part of the questionnaire focused on graduates’ perceptions of curriculum organization; the second part focused on Azjen’s concept of “perceived behavioral control” to investigate graduates’ readiness for clinical practice. Participants were asked to reflect on their learning status against each of the aforementioned six core competencies of the ACGME for physicians when responding to the questions [23].

Because the notion of “student satisfaction” can be regarded as the outcome of a learning process or the requirement that contributes to successful learning,” we included three items in the questionnaire to distinguish the two: “I am provided with sufficient meaningful tasks to acquire ACGME core competencies,” “The training program helps develop my expertise in ACGME core competencies,” and “What I am required to learn is relevant to enhance my core competencies” [4, 24]. The participants responded to these three questions in relation to each of the six ACGME competencies. Therefore, this part of the questionnaire had 18 items.

The theory of planned behavior (TPB), proposed by Fishbein and Ajzen [25], has been used extensively and successfully to investigate the associations between perceived behavioral control and intentions for not only the field of health promotion [26, 27] but also medical education [28–30]. This theory has also been applied systematically to examine and clarify the factors associated with attitude, perceived behavior control, and intention during postgraduate medical training [31]. According to an individual’s desire to reach a goal and the feasibility of achieving that goal, reaching an intended outcome is the core component of effective preparation work [23]. Goals are most likely to be established when the anticipated result is perceived as both desirable and feasible [32]. According to the TPB, feasibility relates to individuals’ perceptions of the difficulty in enacting an intended behavior, that is, perceived behavioral control [33]. To investigate students’ readiness for upcoming clinical practice, we employed two statements to examine each of the six ACGME core competencies (yielding 12 items in total) to assess respondents’ self-efficacy in completing future clinical training [34]. The two statements were as follows: “Based on the medical training I have received so far, I am confident in practices relating to” these listed core competencies (items 19–24), and “For my PGY residency training, I am not worried about practices relating to” these listed core competencies (items 25–30). Items were deliberately worded in positive tones because the use of alternating positive and negative wordings was reported to be confusing [35]. All items are summarized in Table 1.

**Table 1:** Questionnaire items

Please rate your agreement (1 = strongly disagree to 5 = strongly agree) with the following statements
A
I am provided with sufficient meaningful tasks for acquiring expertise in
1 Patient care__
2 Medical knowledge__
3 Practice-based learning and improvement__
4 Interpersonal and communication skills__
5 Professionalism__
6 System-based practice__
The training program helps to develop my expertise in
7 Patient care__
8 Medical knowledge__
9 Practice-based learning and improvement__
10 Interpersonal and communication skills__
11 Professionalism__
12 System-based practice__
What I am required to learn is relevant to
13 Patient care__
14 Medical knowledge__
15 Practice-based learning and improvement__
16 Interpersonal and communication skills__
17 Professionalism__
18 System-based practice__
B
Based on the medical training I have received so far, I am confident in practices relating to
19 Patient care__
20 Medical knowledge__
21 Practice-based learning and improvement__
22 Interpersonal and communication skills__
23 Professionalism__
24 System-based practice__
For my PGY residency training, I am not worried about practices relating to
25 Patient care__
26 Medical knowledge__
27 Practice-based learning and improvement__
28 Interpersonal and communication skills__
29 Professionalism__
30 System-based practice__

### Statistical methods

#### Item analysis and factor analysis

The extreme group design for item analysis was first used to examine the validity and reliability of this questionnaire [36]. Next, a principal component analysis (PCA) of the responses was conducted, and a scree plot analysis was used to determine the minimum number of factors, accounting for a large proportion of correlations between the responses. Measures of internal consistency (Cronbach’s alpha) were evaluated for responses to the statements. A low alpha value can be caused by low item-wise correlations among pairs of items; hence, some items may be deleted to increase the coefficient value [37]. In the development of research instruments, trivial items are commonly removed to improve the alpha value [38–40]. In this study, items with a corrected item-total correlation of > 0.5 were considered acceptable [37]; this value indicates that the items measure the same underlying concept. An exploratory factor analysis using PCA and varimax rotation was conducted to determine the factor structure among the items in the final study. To minimize ambiguity, items were only included in the final version if their factor loadings were > 0.5 and no cross-factor loading of > 0.5 was noted in two or more components.

#### Data analysis

The descriptive results of categorical variables, such as respondents’ sex and clinical training system in medical school, are expressed as the number and percentage of each category. Continuous variables, such as age and perceptions of clinical training, are expressed as the mean ± standard deviation (SD). For univariate analysis, a two-sample hypothesis-testing approach was used to assess differences in the mean value for the perceptions of clinical training of categorical variables. The Pearson correlation coefficient was also used to assess the correlation between continuous variables. A stepwise multiple regression analysis was used to identify predictors of medical students’ preparedness for PGY residency training. The independent variables were sex, age, clinical training system in medical school, and respondents’ perceptions of curriculum organization. *p* < 0.05 was considered significant. All statistical analyses were performed using SPSS version 20.0 (SPSS, Chicago, IL, USA).

## Results

### Descriptive information

The descriptive results are presented in Table 2. In total, 197 of the 315 graduates completed the survey (response rate: 62.5%). The respondents’ mean age was 25.08 years (SD = 1.58); 60.4% of them were men, and 54.8% had graduated from the new 6-year clinical training program.

**Table 2:** Descriptive information of respondents’ demographics data (*N*=197)

**Variables**	**Range**	**Mean ± SD**	**N**	**%**
Age (years)	23 to 34	25.08 ± 1.58	196	
Sex				
Male			119	60.4
Female			78	39.6
Medical school curriculum				
Traditional 7-year curriculum			89	45.2
New 6-year curriculum			108	54.8

### Results of item analysis

Table 3 presents the results of the item analysis of the two investigated scales. The Cronbach’s alpha of Scale A—Perceptions Regarding Curriculum Organization—was 0.945, and all 18 statements had a corrected item-total correlation of > 0.5; these items were reserved for further PCA. One of the 12 statements listed in Scale B—Preparedness for PGY Residency Training—was “Based on the medical training I have received so far, I am confident in practice on medical knowledge” (Item 20). This item had a corrected item-total correlation (0.442) < 0.5 and was thus deleted to improve the Cronbach’s alpha value from 0.912 to 0.913.

**Table 3:** Item analysis for the Perceptions Regarding Curriculum Organization and Preparedness for PGY Residency Training scales

Item Number	Differentiation	Congeniality			Action
	Critical ratio	Item-total Correlation	Corrected Item-total correlation	Cronbach’s α if item deleted	
Scale A^a^					
1	10.344^***^	0.688^***^	0.640	0.943^a^	Reserved
2	8.993^***^	0.666^***^	0.618	0.943^a^	Reserved
3	10.551^***^	0.715^***^	0.675	0.942^a^	Reserved
4	11.820^***^	0.697^***^	0.653	0.942^a^	Reserved
5	11.713^***^	0.736^***^	0.699	0.941^a^	Reserved
6	11.180^***^	0.751^***^	0.714	0.941^a^	Reserved
7	10.251^***^	0.682^***^	0.638	0.943^a^	Reserved
8	7.502^***^	0.571^***^	0.524	0.944^a^	Reserved
9	10.578^***^	0.747^***^	0.709	0.941^a^	Reserved
10	11.088^***^	0.673^***^	0.626	0.943^a^	Reserved
11	10.225^***^	0.737^***^	0.700	0.941^a^	Reserved
12	10.150^***^	0.747^***^	0.706	0.941^a^	Reserved
13	10.664^***^	0.740^***^	0.700	0.941^a^	Reserved
14	9.605^***^	0.688^***^	0.647	0.942^a^	Reserved
15	10.165^***^	0.754^***^	0.719	0.941^a^	Reserved
16	13.238^***^	0.788^***^	0.757	0.940^a^	Reserved
17	10.672^***^	0.777^***^	0.746	0.941^a^	Reserved
18	10.965^***^	0.776^***^	0.740	0.941^a^	Reserved
Scale B^b^					
19	10.683^***^	0.681^***^	0.613	0.906^b^	Reserved
20	8.117^***^	0.529^***^	0.442	0.913^b^	Deleted
21	11.792^***^	0.760^***^	0.714	0.902^b^	Reserved
22	12.042^***^	0.712^***^	0.657	0.904^b^	Reserved
23	10.922^***^	0.692^***^	0.636	0.905^b^	Reserved
24	10.804^***^	0.704^***^	0.643	0.905^b^	Reserved
25	9.150^***^	0.676^***^	0.602	0.907^b^	Reserved
26	9.611^***^	0.672^***^	0.600	0.907^b^	Reserved
27	12.897^***^	0.798^***^	0.744	0.900^b^	Reserved
28	13.947^***^	0.783^***^	0.719	0.901^b^	Reserved
29	14.331^***^	0.783^***^	0.721	0.901^b^	Reserved
30	13.268^***^	0.772^***^	0.709	0.901^b^	Reserved

^a^Scale A: Perceptions Regarding Curriculum Organization; Cronbach’s α = 0.945

^b^Scale B: Preparedness for PGY Residency Training; Cronbach’s α = 0.912

^***^ *p* < 0.001

### Results of PCA

PCA with varimax rotation was conducted separately for both investigated scales. Table 4 presents the factor loadings for each item. In Scale A, 3 of 18 items satisfied the Kaiser–Meyer–Olkin (KMO) criterion with an eigenvalue of > 1 (7.972, 1.220, and 1.017) and accounted for 68.06% of the variance (KMO = 0.906; Bartlett sphericity test result = 0.000). Their eigenvalues were 3.687, 3.598, and 2.924, respectively, rotated using the varimax method (Cronbach’s alpha: 0.876, 0.902, and 0.851, respectively). After varimax rotation, the three components (rotated factors) accounted for 24.580%, 23.989%, and 19.492% of the variance, respectively. These three components were A1 “perceived sufficiency of medical training,” A2 “perceived usefulness of medical training,” and A3 “perceived appropriateness of the educational setting.” Three items (items 10, 13, and 2) were subsequently deleted because their cross-factor loadings were > 0.5 in two or more components.

**Table 4:** Factor loading for the contributing items in the questionnaire

Item Number	Factor loading		
	Factor 1	Factor 2	Factor 3
Scale A^a^	A1	A2	A3
1	0.668		
3	0.737		
4	0.796		
5	0.695		
6	0.741		
7			0.757
8			0.717
9			0.571
11			0.600
12			0.560
14		0.640	
15		0.717	
16		0.741	
17		0.807	
18		0.782	
Scale B^b^	B1	B2	
21		0.847	
22		0.745	
23		0.856	
24		0.831	
25	0.688		
26	0.704		
27	0.837		
28	0.785		
29	0.834		
30	0.842		

^a^Scale A: Perception Curriculum Organization

^b^Scale B: Preparedness for PGY Residency Training

For Scale B, 11 of the original listed 12 items were subjected to further principal factor analysis. The PCA results loaded onto two factors, which together accounted for 70.54% of the variance in the data (KMO = 0.876; Bartlett sphericity test result = 0.000). The eigenvalues of the two components were 3.962 and 3.092 rotated using the varimax method, (Cronbach’s alpha: 0.904 and 0.881, respectively). After varimax rotation, two components—B1 (“unworried about PGY residency training”) and B2 (“confidence in practice”)—accounted for 39.617% and 30.920% of the variance, respectively. One item (Item 19) was deleted because its cross-factor loading was > 0.5 in both components.

### Results of data analysis

Table 5 presents the results of univariate analyses using the t test for categorical variables (sex and clinical training systems) and Pearson’s correlation coefficient for continuous variables. Male graduates had a significantly higher score on both Scale A (58.78 vs. 55.67, *p* = 0.010) and Scale B (33.52 vs. 30.43, *p* = 0.001). The graduates from the new 6-year clinical training system had a significantly lower score on Scale B (30.63 vs. 34.36, *p* < 0.001) but not on Scale A. Age was not significantly correlated with scores in either subscale. The respondents’ Scale A scores demonstrated a significant positive correlation with Scale B scores (Pearson *R* = 0.490, *p* < 0.001).

**Table 5:** Univariate analysis of the scores on Scales A and B

	**Scale A**			**Scale B**		
**Variables**	**Mean ± SD**	**Pearson R**	***p***	**Mean ± SD**	**Pearson R**	***p***
Sex						
Male	58.78 ± 7.81		0.010	33.52 ± 6.20		0.001
Female	55.67 ± 8.68			30.43 ± 6.74		
Medical school curriculum						
Traditional 7-year curricular setting	58.44 ± 8.80		0.169	34.36 ± 6.30		< 0.001
New 6-year curricular setting	56.81 ± 7.81			30.63 ± 6.34		
Age		− 0.043	0.549		0.039	0.586
Scale A		1			0.490	< 0.001

Table 6 presents the results of stepwise multiple regressions of medical students’ preparedness for PGY residency training. In the stepwise regression model (adjusted *R*^*2*^ = 0.469, *p* < 0.001) for graduates’ self-confidence, four factors were included. Factor A1, “perceived sufficiency of medical training” (*R*^*2*^ = 0.411), is the first included in the stepwise regression model, followed sequentially by factor A2, “perceived usefulness of medical training” (△R^2^ = 0.032), sex (△R^2^ = 0.021), and curricular setting (△R^2^ = 0.016). Regarding the graduates’ *unworried state*, two factors were included in the final stepwise regression model (adjusted *R*^*2*^ = 0.205, *p* < 0.001), namely “perceived sufficiency of medical training” (*R*^*2*^ = 0.157) followed by “curricular setting” (△R^2^ = 0.056).

**Table 6:** Stepwise regressions of medical students’ perceptions of preparedness for PGY residency training

	Confidence		Unworried state	
Variables	B	95% CI	B	95% CI
Constant	5.381		8.113	
Demographics				
Age	e		e	
Sex (female vs. male)	− 0.752^**^	− 1.293 to − 0.212	e	
Curriculum (6-year vs. 7-year)	− 0.648^*^	− 1.177 to − 0.118	− 2.287^***^	− 3.510 to − 1.064
Appreciation of Curriculum Organization				
Perceived sufficiency	0.361^***^	0.249 to 0.473	0.548^***^	0.355 to 0.740
Perceived usefulness	0.182^**^	0.070 to 0.294	e	
Perceived appropriateness	e		e	
F	43.805^***^		25.879^***^	
R	0.693		0.462	
R^2^	0.480		0.213	
Adjusted R^2^	0.469		0.205	

e: variable excluded from the regression model

^*^p < 0.05, ^**^p < 0.01, ^***^p < 0.001

## Discussion and conclusion

Studies on medical students’ perceptions of their undergraduate education have focused on students’ evaluations of curriculum quality and their readiness for future clinical practice [5, 14, 41–45]. In the present study, we focused on these two indispensable domains to compare the effectiveness of a 7-year versus a 6-year training program. We investigated whether the curriculum reform resulted in distinct evaluations by students from the two academic training programs. The PCA confirmed the validity of our 25-item questionnaire.

Five items were excluded from the analysis. Two items were removed because of the participants’ inability to distinguish between having confidence in medical knowledge (item 20) and having sufficient medical knowledge (item 2). The respondents also struggled to answer the following two questions: “To what extent is the training for interpersonal communication sufficient (item 10)?” and “To what extent is the teaching of patient care sufficient (items 13 and 19)?” because of their little experience in interpersonal practice and knowledge of primary patient care. Thus, these three items were removed.

The Pearson correlation analysis results also indicated that both main constructs—the perceptions regarding curriculum organization and preparedness for PGY residency training—were moderately correlated with each other.

The t-test results revealed that our graduates from the 6-year program had significantly lower scores for their preparedness for PGY residency training than their counterparts who graduated from the 7-year program. Because of the curriculum reform, the original number of compulsory credits in the medical school where the survey was conducted was reduced from 219 to 199 credits, divided among several clinical learning courses. According to the implementation guidelines for the clinical placement of medical students in the new medical curriculum, the daily working hours for medical clerks may not exceed 12 h [46]. This requirement was absent in the previous 7-year curriculum. Clerks in the 6-year program can have a maximum of three patients in their primary care at each rotated department, whereas clerks in the 7-year program could have up to 10 primary care patients. These protective measures for clinical placement are progressive in terms of social justice and enable clerks to appreciate every aspect of clinical learning. Our results indicated no significant difference in the perceptions regarding curriculum organization between the students in the 6-year and 7-year programs; however, those in the 7-year program reported greater preparedness for residency training. This disparity may be explained by the revised Bloom’s taxonomy of education proposed by Anderson for the four knowledge levels, namely practical knowledge, theoretical knowledge, procedural knowledge, and metacognitive knowledge, the highest level [47]. Students of the 6-year program lacked a 1-year internship, which mostly involves “learning by doing” [48] in the workplace, resulting in a shorter clinical learning period; therefore, students in the 7-year program were able to develop greater confidence in their clinical competency [21]. Other potential factors driving the lower rating of the 6-year curriculum include the challenges associated with transitioning to a new curriculum, available teaching resources, the lack of longer-term follow-up data, or further in-depth qualitative interview results.

This study had some limitations. First, we did not include some factors to investigate whether the differences between the two curricula also resulted in academic performance disparities. Vokes et al. measured the rate of honor grades in clerkships at different medical schools in the United States to examine the utility of clerkship grades in evaluating orthopedic surgery residency applicants and found that a standardized method for grading medical students during clinical clerkships does not exist, resulting in a high degree of interinstitutional variability [49]. Surgery clerkship grades are unreliable for comparing orthopedic surgery residency applicants from different medical schools [49]. However, medical educators in Taiwan lack the ability to specifically identify the cause of differing perceptions or areas needing improvement. Future studies should investigate whether the same situation is applicable to Taiwan.

Second, Newton et al. used factor analysis to explore nursing students’ perceptions of factors related to the clinical learning environment [43]; the results revealed that educational strategies should be developed to sustain a student-centered approach in clinical practice [50]. Therefore, a more comprehensive theoretical framework with comprehensive descriptive items that serves as the basis of the standardized measure of applicant evaluation might be helpful in the future.

Third, the results of the independent *t* test indicated that the male graduates had a significantly higher score on both scales than did the female graduates. The results of stepwise regression also revealed that sex difference significantly correlated with graduates’ readiness for PGY residency training. This might be due to a significant gap between real and perceived preparedness in terms of knowledge and skills among female students. A previous Canadian study indicated that female students’ self-assessment scores were significantly lower than the scores they received from their peers, whereas no significant difference was observed between self-assessment and peer assessment scores for male examinees [51]. American female medical students also reported more anxiety and less self-confidence in their abilities than their male counterparts [52]. Therefore, anxious emotions may also reduce the perceived self-confidence of female students [51, 53]. In another study, female physicians had significantly lower self-reported self-efficacy than their male counterparts [54], negatively affecting the willingness to take on leadership roles in hospitals [33]. Therefore, to avoid sex disparities in career development, female medical students’ learning environment and conditions merit further investigation.

Finally, our cross-sectional questionnaire survey results only reflect the subjective perceptions of medical undergraduates’ regarding the curriculum and preparation for residency training before and after the medical reform. Further quantitative studies with repeated measurements of detailed survey questions or qualitative studies with open-ended interview questions would more comprehensively elucidate students’ perceptions. Because our study was conducted during the transition between the two curricula, the graduates from both undergraduate programs simultaneously participated in PGY residency training. Close monitoring of our ongoing follow-up study is necessary to assess graduates’ objective academic outcomes or clinical performance in the workplace.

## Data Availability

The datasets generated and/or analyzed during the current study are not publicly available due to protection of participant confidentiality. To request the data, please contact the corresponding author.

## Abbreviations

ACGME: Accreditation Council for Graduate Medical Education
KMO: Kaiser–Meyer–Olkin
PCA: Principal component analysis
PGY: Postgraduate-year
SD: Standard deviation
TPB: Theory of planned behavior
